# Signal pathways JNK and NF-κB, identified by global gene expression profiling, are involved in regulation of TNFα-induced mPGES-1 and COX-2 expression in gingival fibroblasts

**DOI:** 10.1186/1471-2164-11-241

**Published:** 2010-04-15

**Authors:** Tove Båge, Johan Lindberg, Joakim Lundeberg, Thomas Modéer, Tülay Yucel-Lindberg

**Affiliations:** 1 Division of Pediatric Dentistry, Department of Dental Medicine, Karolinska Institutet, Huddinge, Sweden; 2 School of Biotechnology, Department of Gene Technology, AlbaNova University Center, Royal Institute of Technology, Stockholm, Sweden

## Abstract

**Background:**

Prostaglandin E_2 _(PGE_2_) is involved in several chronic inflammatory diseases including periodontitis, which causes loss of the gingival tissue and alveolar bone supporting the teeth. We have previously shown that tumor necrosis factor α (TNFα) induces PGE_2 _synthesis in gingival fibroblasts. In this study we aimed to investigate the global gene expression profile of TNFα-stimulated primary human gingival fibroblasts, focusing on signal pathways related to the PGE_2_-synthesizing enzymes prostaglandin E synthases (PGES), as well as the upstream enzyme cyclooxygenase-2 (COX-2) and PGE_2 _production.

**Results:**

Microarray and western blot analyses showed that the mRNA and protein expression of the inflammatory induced microsomal prostaglandin E synthase-1 (mPGES-1) was up-regulated by the cytokine TNFα, accompanied by enhanced expression of COX-2 and increased production of PGE_2_. In contrast, the expression of the isoenzymes microsomal prostaglandin E synthase-2 (mPGES-2) and cytosolic prostaglandin E synthase (cPGES) was unaffected by TNFα treatment. Using oligonucleotide microarray analysis in a time-course factorial design including time points 1, 3 and 6 h, differentially expressed genes in response to TNFα treatment were identified. Enrichment analysis of microarray data indicated two positively regulated signal transduction pathways: c-Jun N-terminal kinase (JNK) and Nuclear Factor-κB (NF-κB). To evaluate their involvement in the regulation of mPGES-1 and COX-2 expression, we used specific inhibitors as well as phosphorylation analysis. Phosphorylation analysis of JNK (T183/Y185) and NF-κB p65 (S536) showed increased phosphorylation in response to TNFα treatment, which was decreased by specific inhibitors of JNK (SP600125) and NF-κB (Bay 11-7082, Ro 106-9920). Inhibitors of JNK and NF-κB also decreased the TNFα-stimulated up-regulation of mPGES-1 and COX-2 as well as PGE_2 _production.

**Conclusion:**

In the global gene expression profile, the enrichment analysis of microarray data identified the two signal transduction pathways JNK and NF-κB as positively regulated by the cytokine TNFα. Inhibition of these TNFα-activated signal pathways reduced the expression of mPGES-1 and COX-2 as well as their end product PGE_2 _in gingival fibroblasts. The involvement of the signal pathways JNK and NF-κB in the regulation of PGE_2 _induced by TNFα may suggest these two pathways as possible attractive targets in the chronic inflammatory disease periodontitis.

## Background

The chronic inflammatory disease periodontitis is characterized by tissue and bone destruction. The current concept of the etiology of periodontitis is that bacterial components of the biofilm initiate the inflammatory cascade, including infiltration of immune cells and production of inflammatory mediators in the periodontal tissue. The initial inflammation, gingivitis, may then develop into a chronic inflammatory state of the gingiva causing destruction of the gingival tissue as well as the alveolar bone resulting in decreased support for the teeth, and ultimately tooth loss [1-3].

Among inflammatory mediators involved in periodontitis, prostaglandin E_2 _(PGE_2_) has been associated with periodontitis as a potent stimulator of bone resorption, and increased PGE_2 _levels have been reported in gingival tissue and gingival fluid from patients with periodontitis [4-9]. Moreover, administration of nonsteroidal anti-inflammatory drugs (NSAID), inhibitors of PGE_2 _production, has been shown to reduce the progression of alveolar bone resorption in patients with periodontitis, implying that PGE_2 _is a key mediator in the pathogenesis of periodontal disease [10,11].

The proinflammatory cytokine TNFα is also reported to play an important part in the pathogenesis of periodontitis [12,13]. For instance, it has been shown that attachment loss is decreased in periodontitis patients after anti-TNF treatment, and subcutaneous administration of recombinant TNFα is demonstrated to exacerbate experimental periodontitis in rats [14,15]. Also, the chronic inflammatory condition rheumatoid arthritis, which shares many characteristics with periodontitis, has been successfully treated using TNFα blockers, further highlighting TNFα as a key inflammatory mediator in chronic inflammatory conditions [16-18]. We have previously shown that TNFα enhances the production of PGE_2 _in gingival fibroblasts, but the intracellular signal pathways regulating PGE_2 _production and PGE_2_-synthesizing enzymes have still not been completely elucidated [4,19].

The biosynthesis of PGE_2 _from membrane lipids is catalyzed by three groups of enzymes acting sequentially. First, phospholipase A_2 _converts membrane lipids to arachidonic acid [20,21]. The cyclooxygenases (COX-1 and COX-2) then convert arachidonic acid to prostaglandin H_2 _[22]. Finally, the third and most recently identified group of isoenzymes is the prostaglandin E synthases (PGE synthases) which catalyze the conversion of COX-derived prostaglandin H_2 _to PGE_2 _[23,24]. Up to date, three PGE synthases have been described: The microsomal and inducible mPGES-1, the constitutively expressed cytosolic cPGES and the most recently discovered microsomal mPGES-2 [25-29]. We have previously reported that mPGES-1 and COX-2 are up-regulated by TNFα and IL-1β in gingival fibroblasts [4,30,31].

The inflammatory mediator PGE_2 _as well as the PGE_2_-regulatory enzymes COX-2 and mPGES-1 have been shown to be up-regulated by inflammatory stimuli such as lipopolysaccharides, IL-1β and TNFα also in other cell types, including gastric fibroblasts, synovial fibroblasts, cardiac fibroblasts and gastric cancer cell lines [32-37]. Various intracellular signaling pathways have been reported to be involved in inflammatory-induced PGE_2 _production and in the expression of PGE_2_-sythesizing enzymes, although these pathways seem to be both cell- and stimulus-specific. For example, in gingival fibroblasts, we have previously reported that the signal pathways PKC and p38 mitogen-activated protein kinase are involved in the regulation of the cytokine-induced COX-2 expression but not in the regulation of mPGES-1 expression [19]. In contrast, these two signal pathways are demonstrated to be involved in cytokine-induced mPGES-1 expression in colonocytes and orbital fibroblasts [19,38,39]. The differences between cell types and between stimuli make it imperative to study the intracellular regulation of mPGES-1 as well as COX-2 using a cell type and stimulus which is relevant to the disease of interest. In periodontal tissue, the most ubiquitous residential cells are gingival fibroblasts. These cells, by producing inflammatory mediators such as cytokines, prostaglandins and proteolytic enzymes, participate in the inflammatory response and contribute to disease persistence [40-44].

In this work, we therefore aim to explore the signal transduction pathways involved in the regulation of PGE_2_-regulating enzymes mPGES-1 and COX-2 in TNFα-stimulated primary gingival fibroblasts through a global gene expression approach, using microarray technology. Our results show that the intracellular signal transduction pathways JNK and NF-κB are involved in the regulation of TNFα-induced mPGES-1 and COX-2 expression, as well as PGE_2 _production, in gingival fibroblasts.

## Results

### Effect of TNFα on protein expression of PGE synthases and COX-2 and production of PGE_2_

**Figure 1:**
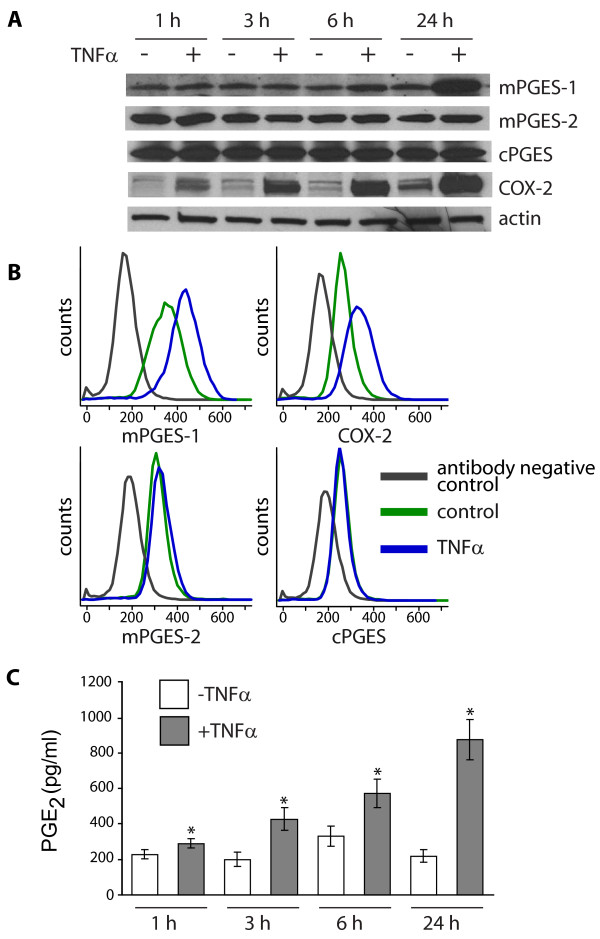
**Effect of TNFα on protein expression of PGE synthases and COX-2 and production of PGE_2_**. **(A) **Gingival fibroblasts were treated with TNFα (20 ng/ml) or without TNFα for the indicated time period. Cells were harvested, total protein was extracted and protein expression of the PGE_2_-synthesizing enzymes mPGES-1, mPGES-2, cPGES and COX-2 as well as the loading control actin was analyzed by western blot. **(B) **Gingival fibroblasts were stimulated with TNFα (20 ng/ml) or cultured without TNFα (control) for 24 h. Protein expression of mPGES-1, mPGES-2, cPGES and COX-2 was investigated by flow cytometry, using specific antibodies. **(C) **Gingival fibroblasts were cultured with or without TNFα (20 ng/ml) for the indicated time period. Levels of PGE_2 _in the culture media were measured by EIA on a Luminex system, and are presented as mean ± s.d. Asterisks (*) indicate a significant difference from untreated cells at the corresponding time point (p < 0.05). The results are representative for all three cell lines and all analyses were performed in triplicates.

We have previously reported that the inflammatory mediator TNFα stimulates PGE_2 _production via the enzymes mPGES-1 and COX-2. In this study we aimed to investigate the signal transduction pathways involved in the regulation of mPGES-1, COX-2 and PGE_2 _in TNFα-stimulated gingival fibroblasts through global gene expression analysis. In agreement with our previous studies [4,30,31], the protein expression of mPGES-1 and COX-2 was up-regulated by the inflammatory mediator TNFα (20 ng/ml), as demonstrated by western blot analysis (Figure 1A). The time-course analysis, using time points 1, 3, 6 and 24 h, revealed that the stimulatory effect of TNFα was strongest at the 24 h time point both for mPGES-1 and COX-2. When comparing these two enzymes, mPGES-1 expression increased starting from the 6 h time point, whereas COX-2 expression time-dependently increased starting from the 1 h time point in response to TNFα stimulation. The expression of COX-2 also increased in cells not stimulated with TNFα in 24 h cultures, as compared to earlier time points. In contrast to mPGES-1 and COX-2, the expression of the PGE synthase isoenzymes mPGES-2 and cPGES was not affected by TNFα in gingival fibroblasts (Figure 1A). The western blot results were also confirmed by flow cytometric analysis, revealing an up-regulation of mPGES-1 and COX-2 expression in 24 h cultures of TNFα-treated cells in contrast to mPGES-2 and cPGES expression (Figure 1B). Moreover, the induction of mPGES-1 and COX-2 expression, in response to TNFα, resulted in a time-dependent increase of PGE_2 _production, as shown in Figure 1C.

### Effect of TNFα on mRNA expression of PGE synthases and COX-2

**Figure 2:**
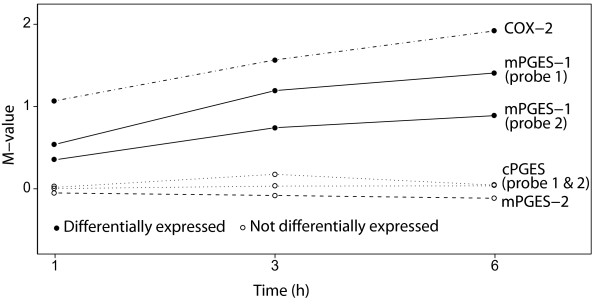
**Effect of TNFα on mRNA expression of PGE synthases and COX-2**. Gingival fibroblasts were treated with TNFα (20 ng/ml) or without TNFα for the indicated time period. Total RNA was extracted from gingival fibroblasts, and mRNA expression of the PGE_2 _synthesizing enzymes mPGES-1, mPGES-2, cPGES and COX-2, was measured by microarray analysis, using specific probes for each enzyme. Two probes for each of the PGE synthase isoenzymes mPGES-1 and cPGES were present on the chip used in the microarray analysis. The y-axis displays the M-value which is the log_2 _of the ratio gene expression of TNFα treated cells/gene expression of control cells. A positive number corresponds to higher expression in the TNFα treated cells. The x-axis corresponds to the different time points. A filled circle denotes a significant differentially expressed gene at the indicated time point whereas an empty circle denotes no significant difference.

The mRNA expression of mPGES-1, mPGES-2, cPGES and COX-2, as measured by oligonucleotide microarray, is seen in Figure 2. The expression of mPGES-1 as well as COX-2 was significantly up-regulated in response to TNFα treatment at the time points indicated (1, 3 and 6 h). On the contrary, the PGE synthase isoenzymes mPGES-2 and cPGES were not differentially expressed at the above-mentioned time points (Figure 2), which is consistent with the protein expression results. The microarray used in this study contained two different probes both for the mPGES-1 and cPGES genes, localized to distinct parts of each mRNA transcript. These probes resulted in similar results, and data from both probes was used in the subsequent analysis.

### Differential expression between TNFα-treated cells and control cells

**Figure 3:**
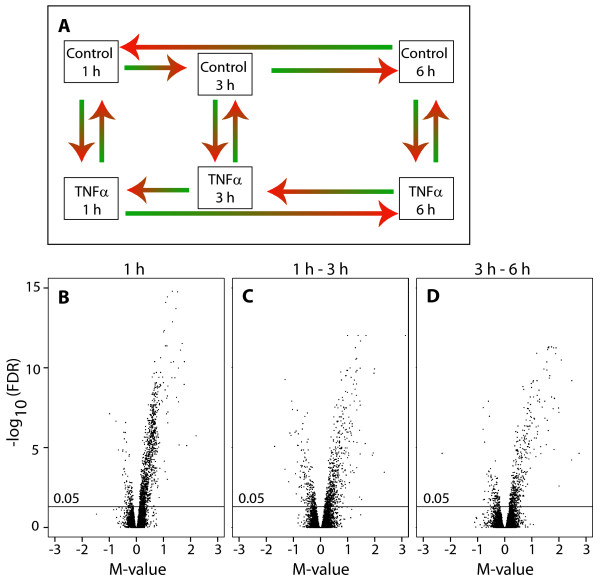
**Microarray experimental design and volcano plots displaying differential expression between TNFα-treated and control cells**. **(A) **For each of the three gingival fibroblast cell lines, cells were treated with TNFα (20 ng/ml) or cultured without TNFα for 1, 3 or 6 h. Samples were labeled and hybridized on oligonucleotide microarrays. Each arrow corresponds to one hybridization where the green sample is labeled with Cy3 and the red sample with Cy5. **(B-D) **The y axis displays the negative false discovery rate (-FDR) on a log_10 _scale and the x axis displays the M-value which is the log_2 _of the ratio gene expression of TNFα treated cells/gene expression of control cells. A positive M-value corresponds to higher expression in the TNFα treated cells. The vertical line signifies a false discovery rate of 0.05, and all genes above it are considered differentially expressed. **(B) **At time point 1 h, 1157 genes were differentially expressed in response to TNFα. For **(C) **and **(D)**, the differentially expressed genes at the later time point were compared with the differentially expressed genes at the earlier time point, to retrieve the genes that were differentially expressed due to time and treatment between those time points. **(C) **Between time points 1 h and 3 h, 796 genes were differentially regulated due to TNFα treatment. **(D) **Between time points 3 h and 6 h, 553 genes were differentially regulated due to TNFα treatment.

In the microarray analysis, the mRNA samples from 1, 3 and 6 h cultures were hybridized according to the experimental design illustrated in Figure 3A. We used a time-course factorial design, which facilitated identification of the genes that were differentially regulated between the time points due to TNFα treatment with optimal statistical efficiency [45]. This design was repeated for all three cell lines included in the study. The majority of the genes that were significantly differentially expressed (differential expression defined as a false discovery rate < 0.05) were up-regulated in the TNFα-treated cells, which is illustrated in the volcano plots in Figure 3B-D. A positive M-value denotes genes up-regulated by TNFα whereas down-regulated genes have a negative M-value. The results showed that 1157 genes were differentially expressed between the TNFα-treated and control fibroblasts after 1 h incubation (Figure 3B). In the comparison between 1 h and 3 h, 796 differentially expressed genes were found (Figure 3C), and 553 genes were differentially expressed between 3 h and 6 h (Figure 3D).

### Gene Ontology analysis of differentially expressed genes

Enrichment analysis was performed to identify overrepresented gene ontology (GO) categories among the differentially expressed genes in the different comparisons. The gene ontology categories represent classes of genes in which more of the individual genes, included in the particular class, are differentially expressed in this data set than would be expected by a random distribution of differentially expressed genes. Complete lists of differentially expressed genes and significant GO categories are presented in the additional material (Additional files 1, 2, 3, 4, 5, 6, 7, 8 and 9).

**Figure 4:**
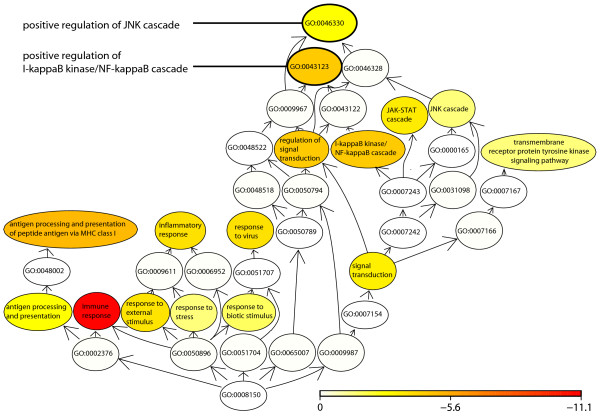
**Gene Ontology analysis of differentially expressed genes**. Gingival fibroblasts were cultured with or without TNFα (20 ng/ml), and cDNA samples were hybridized to an oligonucleotide microarray. Enrichment analysis was performed to identify biological themes among the genes that were differentially expressed in the different comparisons. The plot shows a selection of the gene ontology (GO) categories that were overrepresented among the genes having a significant change in expression between 1 h and 3 h due to TNFα treatment. The color of each node illustrates significance and can be interpreted in the scale bar, which displays the false discovery rate on a log_10 _scale. Arrows indicate the parent to child direction. For clarity, due to space reasons the figure does not include all significant GO categories. Complete lists of significant GO categories for the different comparisons can be found in the additional material (Additional files 2, 5 and 8).

The analysis of the differentially expressed genes at time point 1 h gave a GO profile indicative of TNFα involvement, where significant categories were immune response, regulation, and apoptosis related (Additional file 2). The same types of categories were also significant due to TNFα-treatment between the 1 h and 3 h time points, signifying that genes annotated to those categories were actively regulated between 1 h and 3 h (Figure 4, for a complete list see Additional file 5). Interestingly, positive regulation of two distinct signaling pathways, "positive regulation of I-kappaB kinase/NF-kappaB cascade" and "positive regulation of JNK cascade", was identified by the enrichment analysis in the comparison of 1 h and 3 h of TNFα treatment, as demonstrated in Figure 4. The differentially expressed genes included in these two GO categories are listed in Table 1, together with the genes of the PGE_2_-synthesizing enzymes PGE synthases and COX-2 for comparison of M-values, fold changes and p values. For instance, in the category "positive regulation of I-kappaB kinase/NF-kappaB cascade", the gene for inhibitor of kappa light polypeptide gene enhancer in B-cells, kinase epsilon (IKBKE), which stimulates the dissociation of NF-κB and its inhibitor [46], was found to be up-regulated by TNFα treatment (Table 1). Furthermore, the comparison of 1 h to 6 h gene expression results showing M-values, fold changes and p values for PGE synthases and COX-2 are presented in Table 2.

**Table 1:** PGE_2_-synthesizing enzymes and differentially expressed genes in selected significant GO categories (1-3 h).

Symbol	Gene Name	M-value^a^	Fold Change	p value
*PGE*_2_*-synthesizing enzymes*				
PTGES	microsomal prostaglandin E synthase-1 (probe 1)	0.65	1.6	2.4 × 10^-4^
PTGES	microsomal prostaglandin E synthase-1 (probe 2)	0.39	1.3	0.09^b^
PTGES2	microsomal prostaglandin E synthase-2	-0.03	1.0	1.00^b^
PTGES3	cytosolic prostaglandin E synthase (probe 1)	0.03	1.0	1.00^b^
PTGES3	cytosolic prostaglandin E synthase (probe 2)	0.16	1.1	0.98^b^
PTGS2	cyclooxygenase-2	0.50	1.4	0.63^b^
				
*GO category: Positive regulation of I-kappaB kinase/NF-kappaB cascade*				
APOL3	apolipoprotein L, 3	1.24	2.4	1.2 × 10^-8^
IKBKE	inhibitor of kappa light polypeptide gene enhancer in B-cells, kinase epsilon	0.63	1.5	5.1 × 10^-4^
IKBKE	inhibitor of kappa light polypeptide gene enhancer in B-cells, kinase epsilon	0.50	1.4	3.9 × 10^-4^
FLNA	filamin A, alpha (actin binding protein 280)	-0.23	-1.2	0.03
NUP62	nucleoporin 62 kDa	0.35	1.3	1.0 × 10^-5^
NUP62	nucleoporin 62 kDa	0.37	1.3	9.5 × 10^-3^
TICAM2	toll-like receptor adaptor molecule 2	0.36	1.3	1.6 × 10^-3^
CD40	CD40 molecule, TNF receptor superfamily member 5	0.40	1.3	3.6 × 10^-3^
TLR3	toll-like receptor 3	0.40	1.3	0.01
TFG	TRK-fused gene	0.27	1.2	0.04
F2R	coagulation factor II (thrombin) receptor	-0.22	-1.2	0.05
RIPK2	receptor-interacting serine-threonine kinase 2	1.14	2.2	1.7 × 10^-6^
TNFRSF10B	tumor necrosis factor receptor superfamily, member 10b	0.35	1.3	1.5 × 10^-3^
TNF	tumor necrosis factor (TNF superfamily, member 2)	-0.46	-1.4	2.1 × 10^-3^
SLC20A1	solute carrier family 20 (phosphate transporter), member 1	-1.17	-2.3	1.4 × 10^-3^
TRIM38	tripartite motif-containing 38	0.33	1.3	0.04
BIRC2	baculoviral IAP repeat-containing 2	0.62	1.5	4.8 × 10^-3^
GJA1	gap junction protein, alpha 1, 43 kDa	-0.64	-1.6	0.02
RELA	v-rel reticuloendotheliosis viral oncogene homolog A, nuclear factor of kappa light polypeptide gene enhancer in B-cells 3, p65 (avian)	0.47	1.4	1.6 × 10^-4^
				
*GO category: Positive regulation of JNK cascade*				
EDA2R	ectodysplasin A2 receptor	-0.34	-1.3	1.3 × 10^-3^
TLR3	toll-like receptor 3	0.40	1.3	0.01
TAOK3	TAO kinase 3	-0.35	-1.3	0.01
TAOK3	TAO kinase 3	-0.28	-1.2	9.0 × 10^-3^
HIPK2	homeodomain interacting protein kinase 2	0.40	1.3	3.4 × 10^-5^
TNF	tumor necrosis factor (TNF superfamily, member 2)	-0.46	-1.4	2.1 × 10^-3^

PGE_2_-synthesizing enzymes and differentially expressed genes in selected significant GO categories when comparing 1 h and 3 h of TNFα treatment. ^a^Positive M-values indicate genes up-regulated by TNFα, and negative M-values indicate genes down-regulated by TNFα between the 1 h and 3 h time points. ^b^Not differentially expressed in the comparison between time-points 1 h and 3 h. Differential expression is defined as p < 0.05.

**Table 2:** Differential expression of PGE_2_-synthesizing enzymes when comparing 1 h and 6 h of TNFα treatment.

Symbol	Gene Name	M-value^a^	Fold Change	p value
PTGES	microsomal prostaglandin E synthase-1 (probe 1)	0.87	1.8	6.2 × 10^-7^
PTGES	microsomal prostaglandin E synthase-1 (probe 2)	0.54	1.5	1.8 × 10^-3^
PTGES2	microsomal prostaglandin E synthase-2	-0.06	1.0	0.83^b^
PTGES3	cytosolic prostaglandin E synthase (probe 1)	0.03	1.0	0.78^b^
PTGES3	cytosolic prostaglandin E synthase (probe 2)	0.03	1.0	0.98^b^
PTGS2	cyclooxygenase-2	0.85	1.8	0.03

^a^Positive M-values indicate up-regulated genes, and negative M-values indicate down-regulated genes. ^b^Not differentially expressed in the comparison between time-points 1 h and 6 h. Differential expression is defined as p < 0.05.

**Figure 5:**
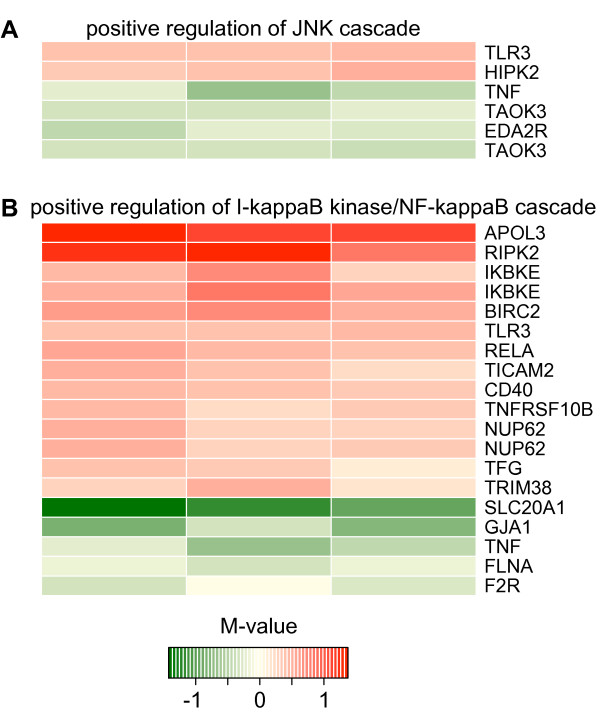
**Heat map**. A heat map demonstrating the relative level of differentially expressed genes between the time points 1 h and 3 h. The color of each gene relates to its M-value (log_2 _ratio of (gene expression of TNFα treated cells/gene expression of control cells)). A positive M-value corresponds to a higher expression in the TNFα treated cells. Gene symbols [91] are used for gene identification. The three columns represent the three cell lines used in the study. **(A) **Heat map for the GO-term GO:0046330 (positive regulation of JNK cascade). **(B) **Heat map for the GO-term GO:0043123 (positive regulation of I-kappaB kinase/NF-kappaB cascade).

When comparing the 3 h and 6 h time points, fewer GO categories were significant than for the 1 h as well as 1 h to 3 h comparisons (26 terms for 1 h, 29 terms for 1 h to 3 h and 10 terms for 3 h to 6 h, see Additional files 2, 5 and 8). Of the two positively regulated signal cascades found in the 1 h to 3 h comparison, only "positive regulation of I-kappaB kinase/NF-kappaB cascade" was significantly changed between 3 h and 6 h. Other significant categories between 3 h and 6 h were mainly immune related (see additional file 8). For the three cell lines used, differential expression of the genes included in the GO categories "positive regulation of JNK cascade" and "positive regulation of I-kappaB kinase/NF-kappaB cascade" in the 1 h to 3 h comparison is also displayed in a heat map in Figure 5.

Since the focus of this study was on signal transduction pathways, we proceeded to further explore the potential role of the two positively regulated intracellular signaling pathways NF-κB and JNK, identified in the 1 h to 3 h comparison, in the regulation of TNFα-induced mPGES-1 and COX-2 expression. For this purpose, we investigated the effect of specific inhibitors of the JNK and NF-κB signal pathways on the expression of mPGES-1 and COX-2 as well as on the phosphorylation of these two pathways.

### Involvement of JNK in the regulation of mPGES-1 and COX-2

**Figure 6:**
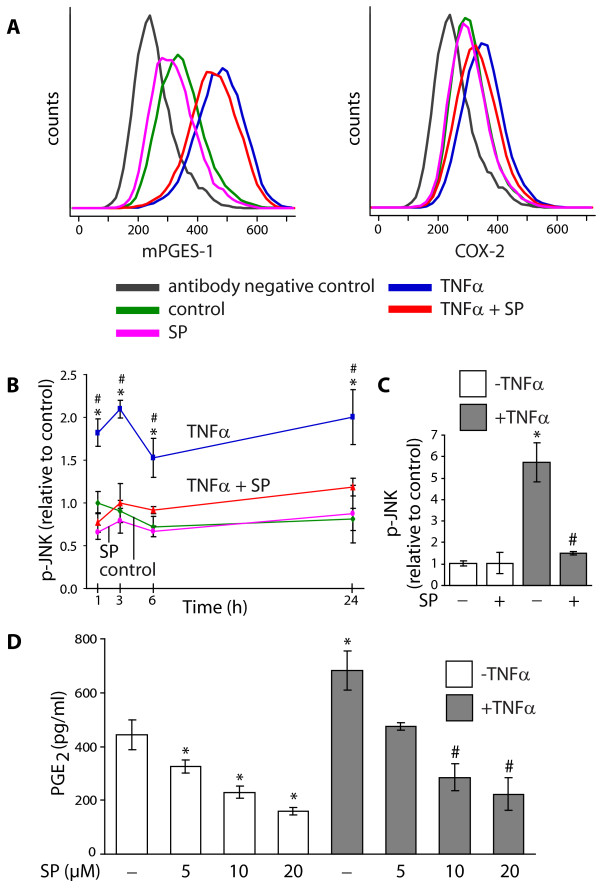
**Involvement of C-Jun N-terminal kinase (JNK) in the regulation of mPGES-1 and COX-2**. **(A) **Gingival fibroblasts were treated with TNFα (20 ng/ml) with or without the JNK inhibitor SP600125 (SP, 10 μM) for 24 h. Expression of mPGES-1 and COX-2 was measured by flow cytometry using specific antibodies. **(B-C) **Gingival fibroblasts were treated with TNFα (20 ng/ml) with or without SP (10 μM) for 1, 3, 6 and 24 h **(B) **or 10 minutes **(C)**. Cells were lysed and total protein was analyzed for phosphorylated JNK (p-JNK) and expressed as relative to control cells at 1 h **(B) **or at start of incubation **(C)**. Data is presented as mean ± s.d. Asterisks (*) indicate a significant difference (p < 0.05) between TNFα-stimulated cells and control cells at each time point, and hash symbols (#) indicate a significant difference (p < 0.05) between TNFα-stimulated cells and cells treated with TNFα in combination with SP at each time point. **(D) **Gingival fibroblasts were cultured with the indicated doses of SP in the absence or presence of TNFα (20 ng/ml) for 24 h. Levels of PGE_2 _in the culture media were measured by EIA using Luminex technology. Data is presented as mean ± s.d. Asterisks (*) indicate a significant difference compared to control cells not treated with TNFα or SP, and hash symbols (#) indicate a significant difference compared to cells treated with TNFα only (p < 0.05). The results are representative for all three cell lines and all analyses were performed in triplicates.

To investigate the involvement of the JNK signal pathway in TNFα-induced mPGES-1 and COX-2 expression and PGE_2 _production, gingival fibroblasts were treated with the specific JNK inhibitor SP600125 (SP) [47]. Treatment of the cells with SP (10 μM) together with TNFα decreased the protein expression of both mPGES-1 and COX-2, the latter to a higher extent, in 24 h cultures compared to cells treated with TNFα only, as demonstrated by flow cytometric analysis in Figure 6A. In addition, SP also decreased mPGES-1 expression in control cells (Figure 6A). To confirm the effect of SP, JNK phosphorylation analysis was performed in 1, 3, 6 and 24 h cultures of fibroblasts stimulated with TNFα in the presence of SP (10 μM) as shown in Figure 6B. The results, expressed as phosphorylated JNK (p-JNK) relative to control cells at 1 h, showed a peak in p-JNK at 3 h of incubation, as compared to unstimulated control cells (Figure 6B). The TNFα-stimulated levels of p-JNK significantly decreased in the presence of SP (10 μM), compared to cells treated with TNFα only, at all time points studied (Figure 6B). Furthermore, the effect of SP on p-JNK was also investigated at an earlier time point, 10 minutes after stimulation (Figure 6C). The results, expressed as phosphorylated JNK (p-JNK) relative to control cells at the start of incubation, showed that the TNFα-stimulated level of p-JNK was decreased to control levels in the presence of SP (Figure 6C). Concurrent with the protein expression results of mPGES-1, the inhibitor (5-20 μM) also decreased the production of PGE_2_, both in TNFα-stimulated cells and in control cells in 24 h cultures, as shown in Figure 6D.

### Involvement of NF-κB in mPGES-1 and COX-2 regulation

**Figure 7:**
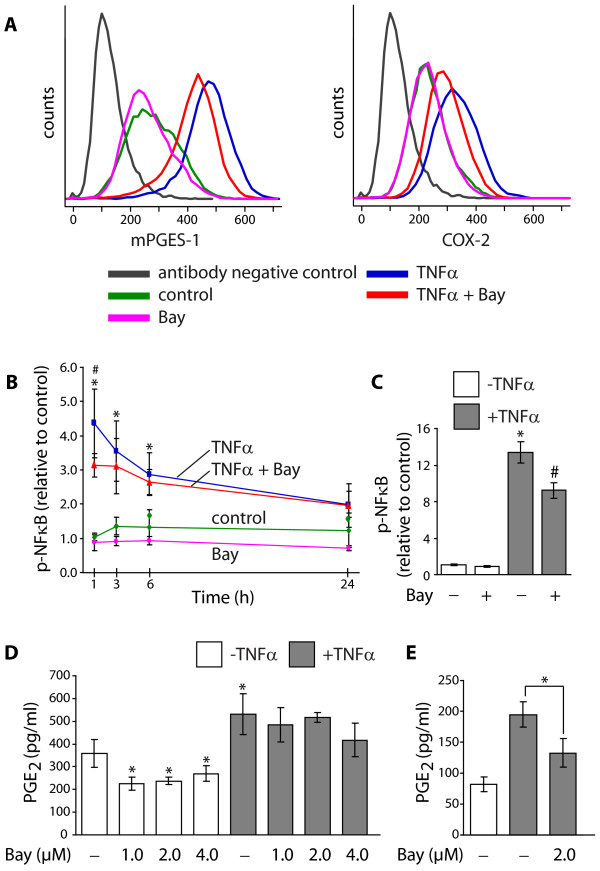
**Involvement of Nuclear Factor-kappaB (NF-κB) in mPGES-1 and COX-2 regulation**. **(A) **Gingival fibroblasts were treated with TNFα (20 ng/ml) with or without the NF-κB pathway inhibitor Bay 11-7082 (Bay, 2.0 μM) for 24 h. Expression of mPGES-1 and COX-2 was measured by flow cytometry using specific antibodies. **(B-C) **Gingival fibroblasts were treated with TNFα (20 ng/ml) with or without Bay (2.0 μM) for 1, 3, 6 and 24 h **(B) **or 10 minutes **(C)**. Cells were lysed and total protein was analyzed for phosphorylated NF-κB p65 (p-NF-κB) and expressed as relative to control cells at 1 h **(B) **or at start of incubation **(C)**. Data is presented as mean ± s.d. Asterisks (*) indicate a significant difference (p < 0.05) between TNFα-stimulated cells and control cells at each time point, and the hash symbols (#) indicate a significant difference (p < 0.05) between TNFα-stimulated cells and cells treated with TNFα together with Bay at each time point. **(D) **Gingival fibroblast were cultured with the indicated doses of Bay in the absence or presence of TNFα (20 ng/ml) for 24 h. Levels of PGE_2 _in the culture media were measured by EIA on a Luminex system, and data is presented as mean ± s.d. Asterisks (*) indicate a significant difference from control cells not treated with TNFα or Bay (p < 0.05). **(E) **Gingival fibroblast were cultured with Bay (2 μM) in the absence or presence of TNFα (20 ng/ml) for 6 h. Levels of PGE_2 _in the culture media were measured by EIA, and data is presented as mean ± s.d. Asterisk (*) indicates a significant difference (p < 0.05). The results are representative for all three cell lines and all analyses were performed in triplicates.

**Figure 8:**
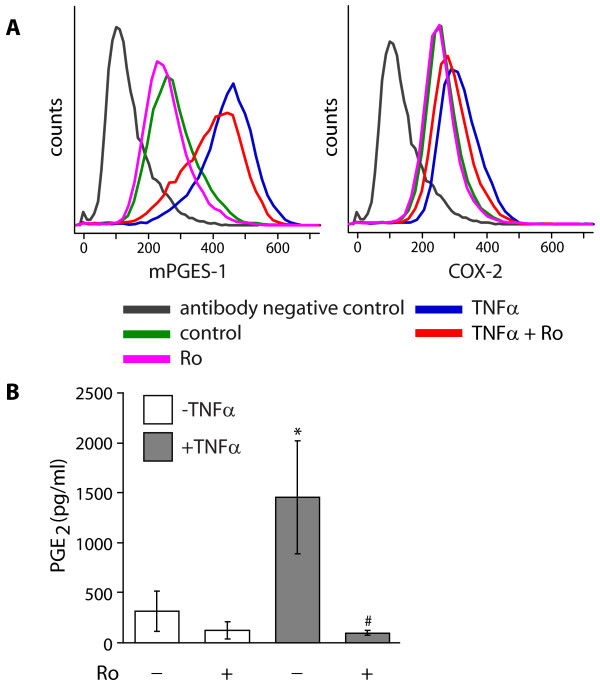
**Inhibition of Nuclear Factor-kappaB (NF-κB) by Ro 106-9920 decreases mPGES-1 and COX-2 expression**. **(A) **Gingival fibroblasts were treated with TNFα (20 ng/ml) with or without the NF-κB activation inhibitor Ro 106-9920 (Ro, 4.0 μM) for 24 h. Expression of mPGES-1 and COX-2 was measured by flow cytometry using specific antibodies. **(B) **Gingival fibroblast were cultured with Ro (4.0 μM) in the absence or presence of TNFα (20 ng/ml) for 24 h. Levels of PGE_2 _in the culture media were measured by EIA, and data is presented as mean ± s.d. Asterisk (*) indicates a significant difference compared to control cells not treated with TNFα or Ro, and the hash symbol (#) indicates a significant difference compared to cells treated with TNFα only (p < 0.05). The results are representative for all three cell lines and all analyses were performed in triplicates.

In order to evaluate the role of NF-κB in the regulation of mPGES-1 and COX-2 expression, the NF-κB pathway inhibitor Bay 11-7082 (Bay) [48] was used in the experiments. Treatment of the cells with Bay (2 μM), decreased the TNFα-stimulated expression of mPGES-1 and COX-2, the latter to a higher extent, in 24 h cultures, as demonstrated by flow cytometric analysis (Figure 7A). In addition, Bay also slightly decreased mPGES-1 expression in control cells (Figure 7A). To confirm the inhibition of NF-κB by Bay, the amount of phosphorylated NF-κB p65 (p-NFκB) in fibroblasts stimulated with TNFα for 1, 3, 6 and 24 h was analyzed using p-NFκB-specific antibodies (Figure 7B). The results, expressed as relative to control cells at the 1 h time point, showed a peak of p-NF-κB at 1 h of incubation with TNFα (Figure 7B). Treatment of the cells with Bay (2 μM) in combination with TNFα significantly decreased p-NF-κB in 1 h cultures, as demonstrated in Figure 7B. The effect of Bay on p-NF-κB was also studied at an earlier time point, 10 minutes after stimulation (Figure 7C). The results, expressed as p-NF-κB relative to control cells at the start of incubation, showed that at the 10 minute time point, the TNFα-stimulated level of p-NF-κB was significantly decreased in the presence of Bay (2.0 μM, Figure 7C). Furthermore, Bay (1-4 μM) reduced the basal production of PGE_2_, compared to control cells in 24 h cultures (Figure 7D). Treatment of TNFα-stimulated cells with Bay (1-4 μM) had no effect on PGE_2 _production in 24 h cultures (Figure 7D), but the inhibitor (2 μM) decreased the TNFα-stimulated PGE_2 _production in 6 h cultures (Figure 7E). To further confirm the involvement of NF-κB in the regulation of mPGES-1, COX-2 and PGE_2_, an additional inhibitor of NF-κB, Ro 106-9920 (Ro) [49], was used. Treatment of the cells with Ro (4 μM), decreased the TNFα-stimulated expression of mPGES-1 and COX-2 in 24 h cultures, as demonstrated by flow cytometric analysis (Figure 8A). Concurrent with the protein expression results, the inhibitor (4 μM) also decreased the production of PGE_2 _in TNFα-stimulated cells in 24 h cultures, as shown in Figure 8B.

## Discussion

PGE_2 _is involved in numerous inflammatory associated diseases including the chronic inflammatory disease periodontitis. The aim of this study was to investigate the global gene expression profile of TNFα-stimulated human gingival fibroblasts with a focus on signal transduction pathways related to the expression of mPGES-1 and COX-2. We used a time-course factorial design for the oligonucleotide microarray hybridizations, which enabled us to statistically discern the interaction effect between the TNFα treatment and time aspect in the experiments studied. To our knowledge, this is the first study investigating the global gene expression profile of TNFα-stimulated gingival cells as an in vitro model of gingival inflammation. In the current study, we demonstrate a TNFα-stimulated up-regulation of mPGES-1 and COX-2 expression, both at mRNA and protein levels, accompanied by enhanced PGE_2 _production. In addition, our microarray results showed a regulation of immune response, apoptosis and signal transduction gene categories in response to TNFα treatment of gingival fibroblasts, including positive regulation of the signal pathways JNK and NF-κB.

The TNFα up-regulated mPGES-1 and COX-2 expression, accompanied by increased PGE_2 _production, is in line with our previously published results on gingival fibroblasts [30]. In contrast to the mPGES-1 and COX-2 enzymes, the expression of the PGE synthases mPGES-2 and cPGES was not affected by TNFα treatment, highlighting the importance of mPGES-1 and COX-2 in the regulation of inflammatory-induced PGE_2 _production. However, when considering the similar kinetics of PGE_2 _production and COX-2 expression, as well as the magnitude of COX-2 induction compared to mPGES-1, COX-2 seems to be the more important enzyme driving the TNFα induced PGE_2 _production in gingival fibroblasts. Increased PGE_2 _production, via induction of the PGE_2_-synthesizing enzymes mPGES-1 or COX-2, has also been reported in other cell types stimulated with TNFα, including synovial cells, chondrocytes and colonocytes [38,50,51].

Various intracellular signaling pathways have been reported to be involved in inflammatory-induced PGE_2 _synthesis, mainly through regulation of COX-2, which is the most widely studied enzyme of the PGE_2 _synthesis chain. In contrast, limited reports exist regarding the regulation of the PGE synthases downstream of COX-2. To further explore the regulation of mPGES-1 and related PGE_2_-synthesizing enzymes in gingival fibroblasts, we here used a global gene expression profiling approach to achieve a broader view of the genes and signal pathways related to the regulation of mPGES-1, in parallel with COX-2, using microarray analysis of TNFα-treated gingival fibroblasts. The effect of TNFα on global gene expression profiles has previously been investigated in synovial fibroblasts, preosteoblasts and HeLa cells, but not in gingival cells [52-56]. With regard to periodontal disease, microarray analysis of gingival tissue has been used in an attempt to define subclasses of periodontitis and to evaluate the effect of periodontal therapy [57,58]. In addition, blood cell gene expression profiling has been performed in subjects with aggressive periodontitis [59]. Concerning gingival fibroblasts, microarray studies have been performed on unstimulated cells from healthy and inflamed gingival tissue, and on IL-1β-stimulated immortalized cells [60,61]. However, a majority of the abovementioned microarray studies on cell cultures use only one time point of RNA analysis after stimulation. In this work, using primary gingival fibroblasts, we employed a time-course factorial design to extract as much relevant information as possible from our data set. Commonly, when using a simpler design for a time series experiment, it becomes difficult to relate the gene expression differences at the different time points to each other. This is greatly facilitated by the use of a time-course factorial design, which allows for the identification of the genes that are differentially regulated between the time points due to TNFα treatment [45].

Our results from the microarray analysis indicated the JNK and NF-κB pathways as possible targets for interrupting the TNFα-induced signal transduction leading to increased expression of the PGE_2_-synthesizing enzymes mPGES-1 and COX-2. Thus, we next further investigated the involvement of JNK and NF-κB in the TNFα-stimulated mPGES-1 and COX-2 expression. By using inhibitors specific for these signal pathways, we could demonstrate that JNK and NF-κB are partly involved in the complex network of intracellular signal transduction pathways leading to increased expression of mPGES-1 and COX-2, as well as PGE_2 _production, in response to the inflammatory cytokine TNFα. To our knowledge, this is the first study pointing out the involvement of JNK in up-regulation of mPGES-1 expression in TNFα-stimulated cells. However, previous studies have shown that mPGES-1 is stimulated by IL-1β through the JNK pathway in cardiac myocytes, cardiac fibroblasts and A549 human lung epithelial cells [36,62,63]. In this study we also show that JNK is involved in TNFα-induced COX-2 expression in gingival fibroblasts, suggesting that JNK-dependent decrease of mPGES-1 may not be the major event whereby the inhibition of this signal pathway exerts its effect on PGE_2 _production. The finding that COX-2 expression was somewhat more influenced by inhibition of the JNK pathway, suggests that the JNK pathway may be more significant for COX-2 induction by TNFα in gingival fibroblasts. The involvement of JNK in TNFα-induced COX-2 expression is in line with results obtained from human alveolar epithelial cells and murine osteoblasts [64,65]. Similarly, IL-1β- and lipopolysaccharide-induced COX-2 has been shown to be mediated through JNK in other cell types [66,67]. In contrast, it has also been reported that COX-2 expression induced by epidermal growth factor is unaffected by JNK inhibition in astrocytes, emphasizing the differences between cell types as well as the inflammatory stimuli used for investigation of signal transduction pathways [68].

The JNK pathway has been implicated in chronic inflammatory disorders such as rheumatoid arthritis and inflammatory bowel disease [69,70]. Moreover, JNK-1 deficiency as well as chemical JNK inhibition has been demonstrated to prevent joint destruction in rodent models of rheumatoid arthritis [69,71,72]. Thus, our novel finding that JNK is partly involved in the regulation of TNFα-induced mPGES-1 expression, which in concert with COX-2 regulates PGE_2 _production in gingival fibroblasts, indicates that this signal pathway may also be of importance in the pathogenesis of periodontitis.

In addition to the JNK pathway, we also found that the NF-κB pathway was involved in the regulation of mPGES-1 and COX-2 in gingival fibroblasts stimulated with TNFα. Our results pointing out the NF-κB pathway in the regulation of mPGES-1 are in line with our previous observations [19]. Induction of mPGES-1 expression using another inflammatory mediator, IL-1β, has been reported to be mediated by NF-κB in A549 cells [73]. In regard to COX-2 regulation, the involvement of the NF-κB pathway in the signal transduction of TNFα-induced COX-2 expression has been observed in other cell types, including endothelial cells and astrocytes [74,75]. Concerning PGE_2 _production, no decrease was observed in TNFα-stimulated PGE_2 _production in 24 h cultures, in contrast to the inhibition observed in 6 h cultures. One reason for the lack of inhibition of PGE_2 _in 24 h cultures by the NF-κB inhibitor Bay, in contrast to mPGES-1 and COX-2 expression, may be a toxic effect of the inhibitor, affecting the release of PGE_2 _although no visual signs of cellular toxicity were observed. However, when using the NF-κB inhibitor Ro, reported to inhibit NF-κB via selective inhibition of TNFα-induced IκBα [49], it decreased the TNFα-stimulated expression of mPGES-1 and COX-2 as well as the production of PGE_2 _in 24 h cultures. Furthermore, both the NF-κB inhibitors Bay and Ro as well as the JNK inhibitor SP decreased the basal expression of mPGES-1 in cells not treated with TNFα, which might be due to slightly raised basal levels of mPGES-1 expression resulting from a lingering effect of the serum present in growth medium before the start of cell culture experiments.

A time-course factorial microarray analysis, like the one performed in this work, yields massive amounts of data. In this study we have focused on the signal transduction aspect, especially the JNK and NF-κB pathways, in order to investigate the regulation of mPGES-1 and COX-2 expression in relation to PGE_2 _production. Inhibition of JNK and NF-κB signal pathways by SP and Ro abolished the production of PGE_2_, although the induction of mPGES-1 and COX-2 by TNFα was not completely abrogated. One explanation for this might be that other enzymes may contribute to the production of PGE_2 _stimulated by TNFα. For instance, it is known that the signal pathways JNK and NF-κB, in addition to the COX-2 and mPGES-1 enzymes, are also involved in the regulation of cPLA_2_, an upstream key enzyme of the PGE_2 _synthesis reported to be induced by TNFα in gingival fibroblasts [76-78]. Another explanation for the strong inhibition of PGE_2 _production, in contrast to the partial reduction of mPGES-1 and COX-2 expression, may be a synergistic effect of the concerted inhibition of these two enzymes, since they are functionally coupled and responsible for the coordinated PGE_2 _synthesis [79]. In addition, one has to be aware that the JNK and NF-κB pathways activated by TNFα may not be entirely responsible for the increased expression of mPGES-1 and COX-2. There are many additional TNFα-regulated genes and pathways involved in the regulation of inflammatory conditions, including PGE_2 _regulatory enzymes, that merit further study, and investigations are ongoing to continue charting the genome-wide effect of TNFα on gingival fibroblasts.

## Conclusions

We here present for the first time a gene expression profiling approach to explore the signal pathways involved in the TNFα-stimulated PGE_2 _production and mPGES-1 and COX-2 expression in gingival fibroblasts. In the global gene expression profile, the enrichment analysis of microarray data identified the two signal transduction pathways JNK and NF-κB as positively regulated by the inflammatory cytokine TNFα. Inhibitors of the JNK and NF-κB pathways reduced the TNFα-stimulated expression of mPGES-1 and to a higher extent COX-2, accompanied by abolished PGE_2 _production. Altogether, the microarray and phosphorylation data provide insight into the regulatory network of signal pathways related to PGE synthase, COX-2 and PGE_2 _production in gingival fibroblasts. The involvement of JNK and NF-κB in the regulation of PGE_2 _production induced by TNFα in gingival fibroblasts suggests these two signal pathways as key elements in the inflammatory-induced PGE_2 _production in gingiva, and also as possible attractive targets in the chronic inflammatory disease periodontitis.

## Methods

### Cell culture of human gingival fibroblasts

Human gingival fibroblasts were established from gingival biopsies obtained from 3 patients, 3 to 12 years of age, with no clinical signs of periodontal disease. The protocol, including the collection of gingival biopsies, was approved by the Regional Ethics Board in Stockholm (Dnr 377/98 and 2007/114-31/4). Minced pieces of gingival tissue were explanted to 25 cm^2 ^Falcon tissue culture flasks containing 5 ml of Dulbecco's Modified Eagle Medium (DMEM) supplemented with 50 units/ml penicillin, 50 μg/ml streptomycin (50 μg/ml) and 5% fetal calf serum (FCS, Invitrogen Life Technologies, Scotland, UK). Gingival fibroblasts were obtained by trypsinization of the primary outgrowth of cells. The cells were grown at 37°C with 5% CO_2 _and routinely passaged using 0.025% trypsin in phosphate-buffered saline (PBS) containing 0.02% EDTA. The fibroblasts used in the experiments proliferated in the logarithmic phase between the 9th and 14th passage. Gingival fibroblasts were seeded in Petri dishes in DMEM supplemented with 5% FCS and cultured for 24 h at 37°C. The cell layers were then rinsed with serum-free DMEM followed by the addition of DMEM with or without the inflammatory mediator TNFα (20 ng/ml, according to previous dose-response studies [19,30]) and in the absence or presence of the inhibitors SP600125 (SP; Sigma-Aldrich, St. Louis, MO, USA), Bay 11-7082 (Bay, Sigma-Aldrich) or Ro 106-9920 (Ro, Tocris Bioscience, Bristol, UK). After different incubation periods, as indicated in the figure legends, culture medium was removed and stored at -20°C for subsequent PGE_2 _determination. The cell monolayer was washed twice with ice-cold PBS and the cells were used for oligonucleotide microarray, flow cytometry or phosphorylation specific luminex analyses.

### RNA extraction

Gingival fibroblasts were seeded and grown as described above. For each of the three cell lines, two 100-mm Petri dishes were used for each treatment. After incubation for 1, 3 or 6 h with or without TNFα (20 ng/ml), the cells were immediately frozen in liquid nitrogen and then stored at -70°C for subsequent isolation of total RNA. Total RNA was isolated from fibroblasts using the commercially available RNeasy kit (Qiagen Inc., CA, USA) and quantified spectrophotometrically at 260/280 nm. The RNA from each pair of dishes with identical cell line and treatment were pooled and treated as one sample throughout further analyses. The RNA quality was assessed using the RNA 6000 Nano LabChip kit of the Bioanalyzer system (Agilent Technologies, Palo Alto, CA, USA).

### Labeling and cDNA synthesis for microarray analysis

The synthesis and labeling of cDNA was performed as previously described by Lindberg et al. 2006[80] Briefly, cDNA was synthesized using random hexamer primers (Operon, Alameda CA, USA) and Superscript III (Invitrogen, San Diego CA, USA). The reaction was terminated and the RNA was hydrolyzed with EDTA and HCl. NaOH was then used to restore pH before proceeding with cDNA purification using MinElute Reaction Cleanup Kit (Qiagen, Hilden, Germany). For washing and elution of the cDNA, 80% EtOH and NaHCO_3 _pH 9 was used instead of supplied buffers due to the presence of TRIS, which would affect the labeling reaction. Labeling was performed using Cy3 and Cy5 mono-reactive esters from Amersham-Biosciences (Little Chalfont, Bucks, UK). The labeling mixture was then purified using MinElute Reaction Cleanup Kit (Qiagen) according to the manufacturer's protocol. A Nanodrop instrument (Nanodrop Technologies, Wilmington, DE USA) was used to confirm labeling success and to measure fluorophore concentrations. The fluorophore concentrations of the labeling reactions were used to balance the sample volumes. To obtain equal fluorophore amounts within each hybridization, a smaller volume of the sample with a higher fluorophore concentration was hybridized with the entire volume of the sample with a lower fluorophore concentration.

### Oligonucleotide microarray

The oligonucleotide microarrays used in this study were printed at the KTH microarray core facility [81]. The 70-mer oligos originate from version 3.03 of Operons Human Genome Oligo Set, and the microarray contains 35344 features representing 28948 Entrez Gene ID:s [82] of which 17972 are unique. Additional information regarding the oligonucleotide microarray can be found in additional files 10 and 11.

### Hybridization

Hybridization was performed as previously described [80]. Briefly, the microarrays were put in a trough and prehybridized with a bovine serum albumin based buffer for 30 minutes in a 42°C water bath. After washing the microarrays, the corresponding Cy3 and Cy5 labeled samples were pooled and hybridization buffer was added to the mixture. Lifter slips (Erie Scientific Company, Shelton, CT, USA) were used to contain the hybridization mixture on the array during hybridization. The arrays were then hybridized over night in a 42°C water bath. After subsequent washing the arrays were immediately scanned.

### Scanning and image processing

Scanning was performed on an Agilent G2565BA scanner (Agilent Technologies, Palo Alto, CA, USA) using a scanner resolution of 10 μm, as previously described [80]. The software GenePix 5.1.0.0 (Axon Instruments, Foster City, CA, USA) was used to extract the raw signals from the TIFF images and to assign each spot an ID.

### Statistical methods and low level analysis of microarray data

The data was analyzed using different packages in the software R [83]. All packages except the KTH package [81] are available in the Bioconductor open source software project for analysis of genomic data [84]. First, the raw data from GenePix was read into R. Thereafter, four filters were used to remove spots with abnormal physical properties, as previously described [80]. On average, 75% of all spots passed the filters for each slide. After filtering, the slides were normalized using print tip Lowess normalization [85]. A linear model in the Bioconductor package LIMMA (Linear models for microarray data) was set up to estimate the M-value and variance for each gene. The M-value is the log_2 _of the fold change (e.g. gene expression of TNFα treated cells/gene expression of control cells). Subsequently, differentially expressed (DE) genes were identified in the different contrasts of interest by using a moderated t-test where information is borrowed across all features present on the microarray to obtain a better variance estimate [86]. A false discovery rate algorithm was then applied to the calculated p values to correct for multiple testing [87]. Thereafter, differentially expressed genes were defined as genes with a q value < 0.05 (the false discovery rate analog of a p value), meaning that the proportion of false positives among the differentially expressed genes was 0.05. The Gene Ontology database was then used to assign functions to genes [88]. Utilizing the Gene Ontology annotation, enrichment analysis was performed to discover biological themes among the differentially expressed genes in the different comparisons [89]. Basically, a conditional hypergeometric test is performed for each GO-term where each gene is counted only in the most specific statistically significant GO-term to decorrelate and facilitate the interpretation of the results. False discovery rate was also used here to correct for multiple testing.

### Design of microarray experiments

The experimental design of the microarray study was set up as a time-course factorial design, to best observe the TNFα-induced gene expression changes over time [45]. A C++ program (provided by the authors of reference [45] on request) was used to determine the exact layout of the design in order to estimate the interaction effect between treatment and time, i.e. genes that are differentially expressed over time, with optimal statistical efficiency [45]. The experimental design is illustrated in Figure 3A, where each arrow represents one hybridization. Thus, each sample was measured four times in this design, and the design was repeated for each of the three cell lines.

### Accession codes

The microarray data set has been deposited at Gene Expression Omnibus (National Center for Biotechnology Information), accession number GSE13903 [90].

### Flow cytometric analysis

Cells were seeded and grown in 60-mm petri dishes as described above. After 24 h of treatment, the cells were collected by trypsinization, and washed three times with PBS. Thereafter, the cells were fixed in 2% paraformaldehyde for 15 min at room temperature (RT) and washed with PBS prior to permeabilization with SAP buffer containing 0.1% Saponin (Sigma-Aldrich) in PBS (15 min, RT). The cells were then incubated with primary antibodies for mPGES-1 (monoclonal mouse, Cayman) mPGES-2 (polyclonal rabbit, Cayman), cPGES (polyclonal rabbit, Cayman) or COX-2 (monoclonal mouse, Cayman) for 40 min in the dark (RT). Antibodies were titrated in preliminary experiments. After washing with SAP-buffer, the cells were incubated with a secondary goat anti-mouse Fluorescein Isothiocyanate-labeled antibody (DakoCytomation, Glostrup, Denmark) or sheep anti-rabbit Phycoerythrin-labeled antibody (Serotec, Oxford, UK) in the dark for 40 min at 4°C. Thereafter, the cells were washed with SAP-buffer, resuspended in PBS and analyzed in a FACSCalibur™ flow cytometer using CellQuest software (Becton & Dickinson, San Jose, CA, USA). For each sample, 10 000 events were acquired and cells were analyzed regarding the expression of mPGES-1, mPGES-2, cPGES or COX-2. The results obtained are illustrated as histograms of cell counts, drawn using the program R, together with the software package rflowcyt [83,84].

### Western blot analysis

Cells were seeded and grown in 60-mm petri dishes as described above. To isolate the total protein, the cells were washed, resuspended in PBS and centrifuged. Thereafter, the pellet was resuspended in 100 μl lysis buffer (10 mM HEPES pH 7.9, 10 mM KCl, 0.1 mM EDTA, 0.1 mM EGTA, 1 mM DTT and protease inhibitors: 1 mM PMSF, pepstatin, aprotinin and leupeptin at 1 μg/ml). The protein concentration of the cell lysates was determined using the Bradford method (Protein Assay; Bio-Rad Laboratories, Hercules, CA, USA) using bovine serum albumin (Sigma-Aldrich) as standard. Equal amounts of the obtained protein were separated by electrophoresis on a 4-15% linear gradient polyacrylamide tris-HCl gel (Bio-Rad) and transferred to a nitrocellulose membrane (Bio-Rad). The membrane was then immersed in blocking buffer (Tris buffered saline with 0.1% Tween 20, TBST, pH 8.0 with 5% defatted dry milk; Bio-Rad) for 1 h at RT and incubated at 4°C over night in blocking buffer with primary antibody diluted 1:200 for mPGES-1 (monoclonal mouse IgG, Cayman), 1:250 for mPGES-2 (polyclonal rabbit IgG, Cayman), 1:150 for cPGES (polyclonal rabbit IgG, Cayman), 1:1000 for COX-2 (monoclonal mouse IgG, Cayman) and 1:20000 for the loading control actin (polyclonal rabbit IgG, Sigma-Aldrich). Following primary antibody incubation, the membranes were washed in TBST and incubated for 1 h in RT with horseradish peroxidase-conjugated secondary antibody, diluted in blocking buffer (1:1000 goat anti-mouse or 1:2000 swine anti-rabbit; Dako Corporation, A/S, Denmark). Finally, the membranes were washed in TBST, developed using enhanced chemiluminescence (ECL) (Amersham Biosciences, Bucks, UK) and exposed to hyperfilm-ECL (Amersham Biosciences).

### Analysis of JNK and NF-κB phosphorylation

Cells were seeded and grown in 60-mm petri dishes as described above. After an incubation period of 1, 3, 6 or 24 h, the cells were scraped in PBS and centrifuged. The pellet was then resuspended in the lysis solution of the Bio-Plex Cell Lysis Kit (Bio-Rad) and frozen at -20°C. The samples were then thawed and centrifuged at 4500 g for 20 min at 4°C, and the supernatant was collected. The lysate protein concentration was determined using the Bradford method (Protein Assay; Bio-Rad) with bovine serum albumin (Sigma-Aldrich) as standard, followed by addition of an equal volume of assay buffer from the Bio-Plex phoshpoprotein detection kit (Bio-Rad). The samples were frozen in -20°C until determination of the amount of phosphorylated JNK (T183/Y185) or NF-κB p65 (S536) using Luminex technology on a Bio-Plex Suspension Array System (Bio-Rad) with the Bio-Plex phoshpoprotein detection kit (Bio-Plex, Bio-Rad) according to the manufacturer's instructions.

### Prostaglandin E_2 _determination

The amount of PGE_2 _in the culture media was determined using Luminex technology on a Bio-Plex Suspension Array System (Bio-Rad) using a commercially available enzyme immunoassay (EIA) kit (Cayman), and a conventional EIA kit (Cayman).

### Statistics for non-microarray analyses

All non-microarray experiments were analyzed in triplicates and reproducible data representing one of at least three independent experiments is demonstrated. Results are expressed as the mean ± s.d. Student's t test (two-tailed) was used in the statistical analysis and p values less than 0.05 were considered statistically significant.

## Authors' contributions

TB participated in conceiving and designing the study, carried out all experimental work and drafted the manuscript. JLi performed the microarray and bioinformatic data analyses and participated in manuscript drafting and study design. JLu participated in designing the study and in editing the manuscript. TM participated in manuscript editing and scientific discussions. TL participated in conceiving and designing the study, as well as in manuscript drafting and editing. All authors read and approved the final manuscript.

## Supplementary Material

Additional file 1 **List of differentially expressed genes at 1 h of TNFα stimulation**. Genes that were differentially expressed in gingival fibroblasts due to TNFα treatment at the 1 h time point. Click here for file

Additional file 2 **List of significant Gene Ontology (GO) categories at 1 h of TNFα stimulation**. Significant GO categories due to TNFα stimulation at the 1 h time point, containing the name and number of each GO category for reference to the different worksheets of Additional file 3. Click here for file

Additional file 3 **Complete lists of differentially expressed genes in the significant GO categories at 1 h of TNFα stimulation**. The differentially expressed genes of each significant GO category are listed on a separate worksheet, labeled with the GO category number. Click here for file

Additional file 4 **List of differentially expressed genes between 1 and 3 h of TNFα stimulation**. Genes that were differentially expressed in gingival fibroblasts due to TNFα treatment between the 1 h and 3 h time points. Click here for file

Additional file 5 **List of significant Gene Ontology (GO) categories between 1 and 3 h of TNFα stimulation**. Significant GO categories due to TNFα stimulation between the 1 h and 3 h time points, containing the name and number of each GO category for reference to the different worksheets of Additional file 6. Click here for file

Additional file 6 **Complete lists of differentially expressed genes in the significant GO categories between 1 and 3 h of TNFα stimulation**. The differentially expressed genes of each significant GO category are listed on a separate worksheet, labeled with the GO category number. Click here for file

Additional file 7 **List of differentially expressed genes between 3 and 6 h of TNFα stimulation**. Genes that were differentially expressed in gingival fibroblasts due to TNFα treatment between the 3 h and 6 h time points. Click here for file

Additional file 8 **List of significant Gene Ontology (GO) categories between 3 and 6 h of TNFα stimulation**. Significant GO categories due to TNFα stimulation between the 3 h and 6 h time points, containing the name and number of each GO category for reference to the different worksheets of Additional file 9. Click here for file

Additional file 9 **Complete lists of differentially expressed genes in the significant GO categories between 3 and 6 h of TNFα stimulation**. The differentially expressed genes of each significant GO category are listed on a separate worksheet, labeled with the GO category number. Click here for file

Additional file 10 **KTH HUM 34k Oligo Microarray**. Additional information concerning the oligonucleotide microarray used in this study. Click here for file

Additional file 11 **Oligo Microarray genelist**. Genelist for the oligonucleotide microarray, including probe sequences and accession numbers. Click here for file
