# Supplementary material for: Signal pathways JNK and NF-κB, identified by global gene expression profiling, are involved in regulation of TNFα-induced mPGES-1 and COX-2 expression in gingival fibroblasts
Source: BMC Genomics. 2010 Apr 15;11:241. doi: 10.1186/1471-2164-11-241 (PMC2873473; doi:10.1186/1471-2164-11-241)
Supplement: Additional file 10 — KTH HUM 34k Oligo Microarray. Additional information concerning the oligonucleotide microarray used in this study. [file 1471-2164-11-241-S10.DOC]

KTH HUM 34k **Oligo Microarray**

Homo Sapiens, Operon ver 3.0

Version 3.0 of the Human Genome Oligo Set contains 34,580 70mer probes representing 24,650 genes and 37,123 gene transcripts. The design is fully based on the Ensembl (<http://www.ensembl.org/>) Human 13,31 Database and Human Genome Sequencing Project and directly deals with alternative splicing variants using common, partial common, or individual transcript oligos. The oligo set comes with complete annotation including Gene Ontology (GO), oligo chromosome coordinates, comparative genome analysis, and other functional annotation (InterPro, Online Mendelian Inheritance in Man [OMIM] disease, and protein family). For our probe design we use state-of-the-art methodology and proprietary software. An amino linker is attached to the 5`end of each oligo.

Array manufacturing

The 34,580 70mer probes were dissolved in 30% DMSO to a concentration of 18 µM. The microarrays were printed with a QArray2 (Genetix) instrument with 48 K2805 pins (Genetix) on Ultra GAPS slides (Corning). The 34,580 longmer probes were spotted in a 28x28 pattern within each block and with a feature center-to-center distance of 155 µm. The quality of the spotted slides was assessed by staining with Syto61 (Molecular Probes) and with hybridization with random nonamers. The slides were UV cross-linked at 150 mJ/cm^2^.
